# Ligustrazine for the Treatment of Unstable Angina: A Meta-Analysis of 16 Randomized Controlled Trials

**DOI:** 10.1155/2016/8617062

**Published:** 2016-04-26

**Authors:** Suman Cao, Wenli Zhao, Huaien Bu, Ye Zhao, Chunquan Yu

**Affiliations:** ^1^Graduate School, Tianjin University of Traditional Chinese Medicine, Tianjin 300193, China; ^2^Department of Neurology, Nankai Hospital, Tianjin Academy of Integrative Medicine, Tianjin 300100, China; ^3^Department of Chinese Medicine, Tianjin University of Traditional Chinese Medicine, Tianjin 300193, China; ^4^Department of Clinical Research, Nankai Hospital, Tianjin Academy of Integrative Medicine, Tianjin 300100, China; ^5^Editorial Department, Tianjin University of Traditional Chinese Medicine, Tianjin 300193, China

## Abstract

Ligustrazine is a principal ingredient of chuanxiong. Concerns regarding the evaluation of the effectiveness of ligustrazine in the treatment of UA have resulted in a meta-analysis combined with recent clinical evidence. Seven computer databases that included the China hospital knowledge database (CHKD), Wanfang Med Online, the Chinese medical journal database (CMJD), PubMed, Cochrane, Embase (Ovid), and Medline (Ovid) were systematically searched. We included randomized controlled trials and quasi-randomized controlled trials. Our systematic review identified 16 RCTs that met our eligibility criteria. Ligustrazine combined with conventional medicine was associated with an increased rate of marked improvement in symptoms and an increased rate of marked improvement of ECG compared with conventional Western medicine alone. Additionally, the use of ligustrazine was associated with significant trends in the reduction of the consumption of nitroglycerin and the level of fibrinogen when compared with conventional Western medicine alone. No firm results were found between the intervention and the control method groups in the reduction of the time of onset or the frequency of acute attack angina due to the high level of heterogeneity. In conclusion, our meta-analysis found that ligustrazine was associated with some benefits for people with unstable angina.

## 1. Introduction

United Nations member states have agreed to reduce premature cardiovascular disease (CVD) mortality 25% by 2025. However, CVD is the major cause of death worldwide which is almost a third of all deaths globally in 2013 [1]. In low and middle income countries (LMIC), the situation is not optimistic similarly. The greatest burden of CVD is approximately 80% of cardiovascular deaths occurring in LMIC [2]. The most of CVD deaths were from coronary heart disease (CHD) [3]. Unstable angina is a common manifestation of this disease. The three principal presentations of UA include rest angina, new-onset severe angina, and increasing angina [4]. Unstable angina is a crucial phase of coronary heart disease with widely variable symptoms and prognoses [5]. Thoracic pain may mark the onset of acute myocardial infarction. It typically occurs at rest and has a sudden onset, sudden worsening, and stuttering recurrence over days and weeks. Unstable angina which is a potentially life-threatening event is relatively more harmful than stable angina pectoris [6].

The objective of UA treatment is the improvement of symptoms, the relief of the progress of the disease, and the prevention of cardiovascular events, particularly myocardial infarction and death [7, 8]. Recently, conventional medicine has consisted of antiplatelet agents, anticoagulant agents, nitrates, beta-adrenergic blockers, calcium channel blockers, and inhibitors of the renin-angiotensin-aldosterone system [9]. Although these treatments are widely used in the acute relief of secondary angina pectoris and the long-term prophylactic management of angina pectoris, chuanxiong might also be useful for UA and for increased safety. Therefore, we contrasted chuanxiong with conventional medicine in this meta-analysis.

Traditional Chinese Medicine (TCM) is the result of Chinese civilization over 3000 years. The Chinese herb chuanxiong belongs to the Umbelliferae family [10]. A book named Shen Nong Ben Cao Jing, which was published 2000 years ago, has been the original and existing writing record about chuanxiong. Ligustrazine is a principal ingredient of chuanxiong. It has been shown to play a critical role in cardiovascular treatments, mediated by inhibition of Ca^2+^ influx and by the release of intracellular Ca^2+^ [11, 12]. It significantly inhibits L-type calcium current in a concentration-dependent manner to make vasodilatory effect, to improve the situation of myocardium ischemia [13, 14]. It also suppressed calcium transient and contraction in rabbit ventricular myocytes under physiological and pathophysiological conditions [15]. Besides, ligustrazine improves attenuation of oxidative stress. Treatment by ligustrazine decreased reactive oxygen species (ROS) production and enhanced cellular glutathione (GSH) levels [16]. Ligustrazine treatment partially restored superoxide dismutase1 (SOD1) activity [17], increasing in NO production [18]. Recently, the oxidative stress has been shown to play a critical role in atherogenesis (AS). The PPAR signal pathway is involved in the molecular mechanism of ligustrazine in the treatment of AS [19]. Although pharmacology research might indicate the cardiovascular protective effects of ligustrazine, the specific outcomes of the effectiveness of ligustrazine have not been elucidated. Therefore, this meta-analysis combined recent clinical evidence to evaluate the effectiveness of ligustrazine in the treatment of UA.

## 2. Methods

### 2.1. Search Strategy

The group systematically searched seven computer databases that included the China hospital knowledge database (CHKD), Wanfang Med Online, the Chinese medical journal database (CMJD), PubMed, Cochrane, Embase (Ovid), and Medline (Ovid). The index words were the following: chuan*∗*xiong, chuanxiong rhizome, Ligusticum wallichii, ligustilide, cnidilide, cnidiumlactone, sedanolide, senkyunolide, ligustrazine, tetramethylpyrazine, chuan*∗*xiong extract, Senkyunone, unstable angina, randomized, controlled trials, controlled clinical trials, and random.

### 2.2. Eligibility Criteria

#### 2.2.1. Types of Studies

We included randomized controlled trials and quasi-randomized controlled trials.

#### 2.2.2. Types of Interventions and Participants

Types of interventions and participants are as follows: (1) Participants who were diagnosed with UAP according to the American College of Cardiology Foundation/American Heart Association (ACCF/AHA) Guidelines for the Diagnosis and Management of Patients with Unstable Ischemic Heart Disease [20]; (2) the International Society and Federation of Cardiology/World Health Organization (ISFC/WHO) guideline [21]; (3) the Chinese Society of Cardiology (CSC) guidelines [22]; (4) other criteria; (5) the included trials designed to compare the effectiveness and safety of chuanxiong with conventional medicine and conventional medicine alone.

#### 2.2.3. Types of Outcomes Measures

Cardiovascular events (CEs) including acute myocardial infarction (AMI) and angina pectoris were the outcome measures. The improvement in the angina symptoms (IAS) and electrocardiogram (IECG) results were used as the outcome measures. Moreover, the lack of improvement or worsening of angina symptoms (NIWAS) and the lack of improvement or worsening of ECG (NIWECG) were used as the outcome measures. Angina onset time (AOT), seizure frequency (SF), reduction in nitroglycerin use (RNU), and the level of fibrinogen (FIB) were also included.

#### 2.2.4. Definitions of Improvements of Symptom and ECG

Compared with the basic improvement in angina symptoms, the improvement of symptom involves that frequency and duration of feeling angina chest pain should be reduced at least 50%. Improvement of ECG should be achieved with at least 0.05 mv at ST segment in ECG compared with basic improvements in ECG [23].

#### 2.2.5. Adverse Events

Adverse events are death, life-threatening events, crippling, disabling, teratogenic effects, requiring special events, and hospitalization.

### 2.3. Data Extraction and Quality Assessment

The qualities of the data were assessed by two independent researchers. Each trial identified in the search was assessed for gender, age, design, diagnosis, standards for the participants, interventions, and outcome measures. Any disagreement between the researchers regarding each trial was resolved by consulting a third researcher. Duplicate studies and records were excluded by screening the titles and abstracts. All remaining articles were screened by examining the full text. The qualities of the trials included in this study were evaluated by each researcher according to the Systematic Reviews of Interventions on Cochrane Handbook, version 5.1.0 [24].

### 2.4. Statistical Analysis

We used RevMan 5.3 (review manager) as provided by the Cochrane collaboration to perform the meta-analyses of the database. Dichotomous data were evaluated with the risk ratios (RRs), and continuous outcomes were evaluated with the mean differences (MDs); for both, the 95% confidence intervals (CIs) and forest plots were applied. The chi-squared test and the* I*-squared statistic were used to assess the heterogeneity. For the studies that did not report statistical heterogeneity (*P* > 0.1,* I*-squared < 25%), a fixed-effect model was used to pool the results. In contrast, the heterogeneity was assessed, and the subgroup analyses that produced the heterogeneity were accounted for. If the studies had statistical heterogeneity that did not have clinical heterogeneity, a random-effect model was used. For the studies with extensive heterogeneity or obvious clinical heterogeneity, descriptiveness analyses were used.

## 3. Results

### 3.1. Description of the Included Trials

**Figure 1:**
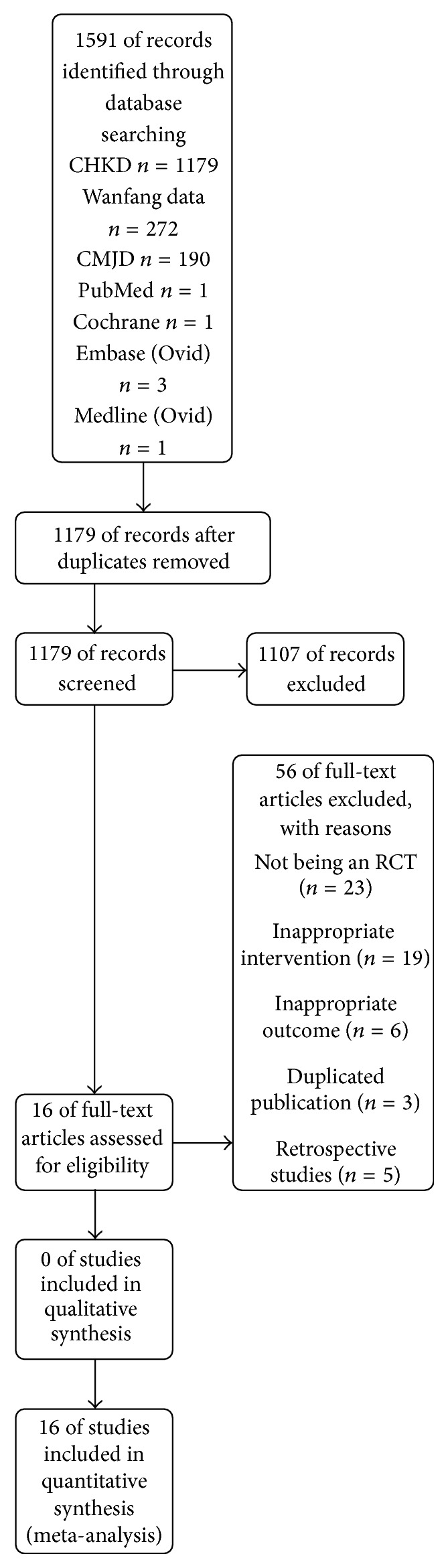
Flow diagram of included and excluded studies.

A total of 1591 trials were identified by database searching and other sources. After examination of duplicates, 1179 trials remained. Proceeding, we excluded 1107 trials. Based on reads of the full articles, 16 RCTs were included according to the eligibility criteria and exclusion criteria. All of these studies were published in Chinese. The literature search and a flowchart of the selection are provided in Figure 1.

All 16 of the included trials were RCTs, and all of the trials recruited participants for the treatment of unstable angina pectoris with chuanxiong in combination with conventional medicine versus conventional medicine. The majority of the studies used the improvement of symptoms and ECG as the outcome measures. Among the studies, five mentioned fibrinogen as an outcome. The time of onset and seizure frequency were also reported in three studies. Reductions in nitroglycerin use were reported in two studies. Cardiovascular events were reported in one study.  Table 1 summarizes the characteristics of these original studies.

### 3.2. The Effect of Ligustrazine

#### 3.2.1. The Rate of Cardiovascular Events

**Figure 2:**
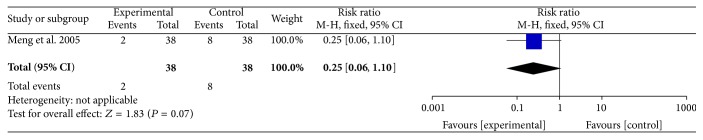
The rate of cardiovascular events and the incidence of angina relapse after 4 weeks.

**Figure 3:**
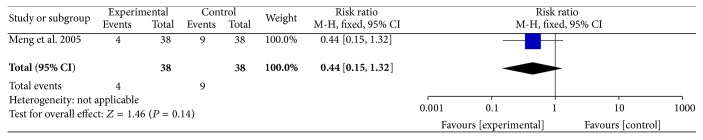
The incidence of angina relapse after 12 weeks.

**Figure 4:**
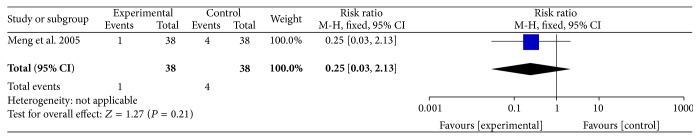
The incidence of AMI relapse after 12 weeks.

A single study showed that ligustrazine was no better or worse at reducing cardiovascular events, including the incidence of angina relapse after four weeks (RR = 0.25, 95% CI (0.06–1.10)) (Figure 2), the incidence of angina relapse after 12 weeks (RR = 0.44, 95% CI (0.15–1.32)) (Figure 3), or the incidence of AMI relapse after 12 weeks (RR = 0.25, 95% CI (0.03–2.13)) (Figure 4). None of the participants relapsed into AMI after four weeks.

#### 3.2.2. Rate of Symptom Improvement

**Figure 5:**
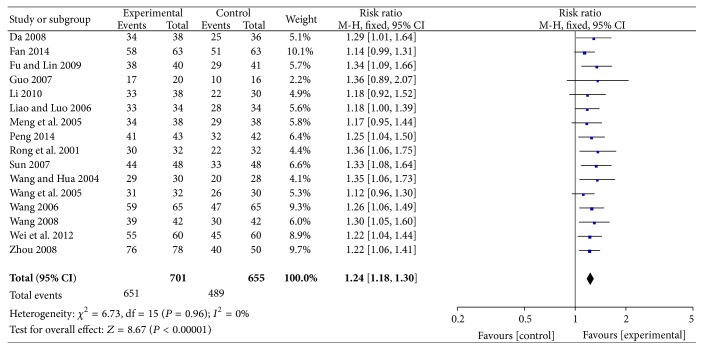
Rate of marked improvement of symptoms.

The rates of symptom improvement were reported in 16 RCTs that involved 1356 participants. All of these studies reported improvements in angina symptoms with ligustrazine compared with conventional medicine. Some of these studies reported evidence that ligustrazine improved angina symptoms (RR = 1.24, 95% CI; 1.18, 1.30). There was no heterogeneity among the 16 studies (*P* = 0.96,* I* ^2^ = 0%) (Figure 5).

#### 3.2.3. Rates of No Improvement or Worsening of Symptoms

**Figure 6:**
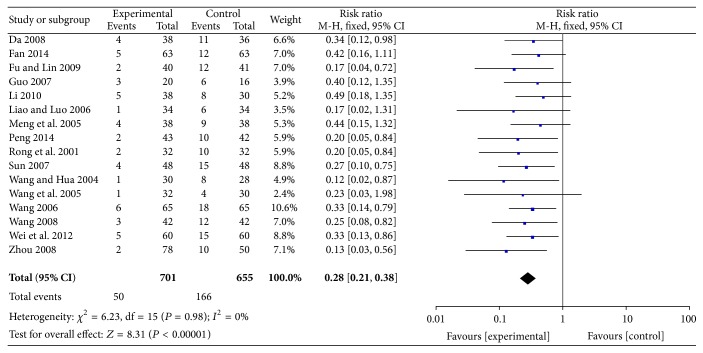
Rate of no improvement or worsening of symptoms.

The rates of no improvement or worsening of symptoms were reported in 16 RCTs involving 1356 participants. Some of the evidence indicated that ligustrazine reduced the number of people with rates of no improvement or worsening of symptoms (RR = 0.28, 95% CI (0.21, 0.38)). There was no heterogeneity among the 16 studies (*P* = 0.98,* I* ^2^ = 0%) (Figure 6).

#### 3.2.4. Rate of Marked Improvement in ECG

**Figure 7:**
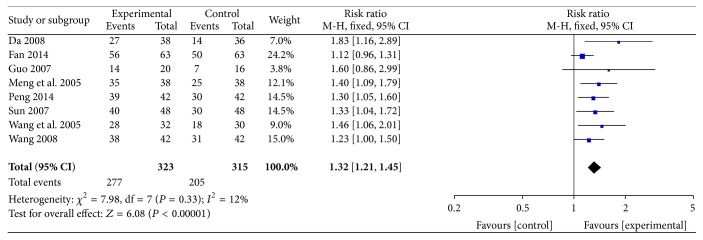
Rate of marked improvement of ECG.

The improvement in ECG was reported in eight RCTs involving 638 participants. All of these studies reported improvements in ECG with ligustrazine compared with conventional medicine. Some evidence indicated that ligustrazine improved ECG (RR = 1.32, 95% CI (1.21, 1.45)). There was no heterogeneity among these eight studies (*P* = 0.33,* I* ^2^ = 12%) (Figure 7).

#### 3.2.5. Rate of No Improvement or Worsening of ECG

**Figure 8:**
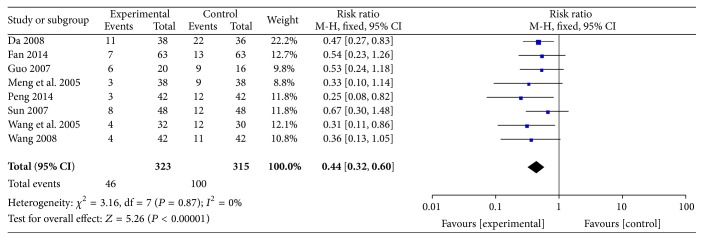
Rate of no improvement or worsening of ECG.

The rates of no improvement or worsening of ECG were reported in eight RCTs involving 638 participants. Ligustrazine reduced the number of people who exhibited no improvement or worsening of ECG (RR = 0.44, 95% CI (0.32, 0.60)). There was no heterogeneity among these eight studies (*P* = 0.87,* I* ^2^ = 0%) (Figure 8).

#### 3.2.6. Time of Onset

**Figure 9:**
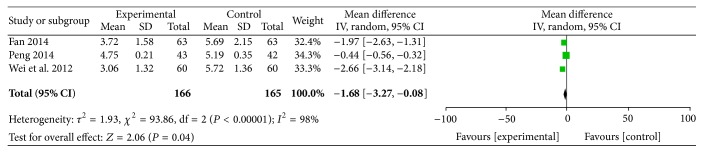
Time of onset.

The time of onset was reported in three RCTs involving 331 participants. All of these studies reported the times of onset for the comparisons of ligustrazine with conventional medicine (MD = −1.68, 95% CI (−3.27, −0.08)). There was a high level of heterogeneity among these three studies (*P* < 0.00001,* I* ^2^ = 98%) (Figure 9).

#### 3.2.7. Frequency of Acute Attack Angina

**Figure 10:**
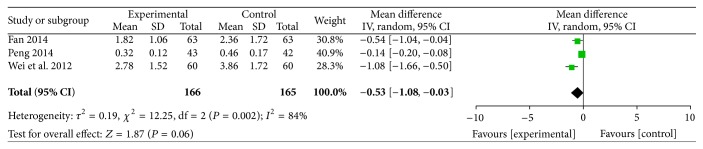
Frequency of acute attack angina.

The frequency of acute attack angina was reported in three RCTs involving 331 participants, and these studies compared the frequency of acute attack angina between ligustrazine and conventional medicine (MD = −0.53, 95% CI (−1.08, −0.03)). There was heterogeneity (*P* = 0.002,* I* ^2^ = 84%) (Figure 10).

#### 3.2.8. Consumption of Nitroglycerine

**Figure 11:**
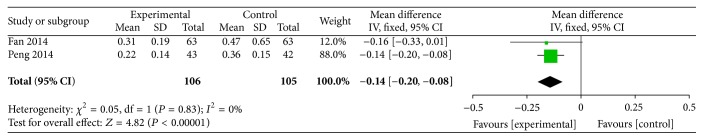
Consumption of nitroglycerine.

Consumption of nitroglycerine was reported in two RCTs involving 211 participants, and these studies reported the comparisons of nitroglycerine consumption between ligustrazine and conventional medicine. Strong evidence revealed that ligustrazine reduced the consumption of nitroglycerine (MD = −0.14, 95% CI 95% (−0.20, −0.08)). There was no heterogeneity (*P* = 0.83,* I* ^2^ = 0%) (Figure 11).

#### 3.2.9. Level of Fibrinogen

**Figure 12:**
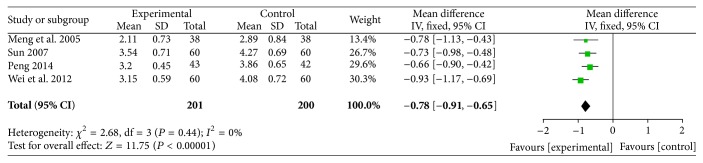
Level of fibrinogen.

The level of fibrinogen was reported in five RCTs involving 437 participants, and all of these studies reported the levels of fibrinogen comparing ligustrazine with conventional medicine. Some evidence revealed that ligustrazine reduced the level of fibrinogen (MD = −0.68, 95% CI (−0.9, −0.46)). There was heterogeneity among these five studies (*P* = 0.03,* I* ^2^ = 64%). We rejected one study for high levels of heterogeneity. The results revealed that ligustrazine reduced level of fibrinogen (MD = −0.78 95% CI (−0.91, −0.65)). There was no heterogeneity among the four included studies (*P* = 0.44,* I* ^2^ = 0%) (Figure 12).

#### 3.2.10. Adverse Events

There were no recorded severe adverse events.

### 3.3. Methodological Qualities of the Included Trials

The risks of seven biases among the 16 trials were evaluated, including random sequence generation, allocation concealment, blinding of participants and personnel, blinding of outcome assessment, incomplete outcome data, selective reporting, and other biases according to the criteria in the Cochrane Handbook for Systematic Reviews [24]. All of the studies described correct randomization methods. There was only one trial with blinding of participants and personnel and blinding of outcome assessment, and nearly all of the trials failed to mention allocation concealment, the blinding of the participants and personnel, and the blinding of outcome assessments. The methodological qualities of the included trials are summarized in Table 2.

### 3.4. Funnel Plot of Publication Bias

**Figure 13:**
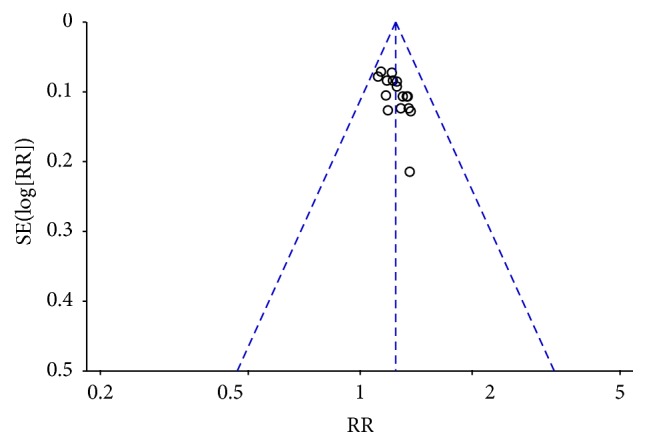
Funnel plots.

The research team used a funnel plot to evaluate the publication biases of all of the included studies, and this plot is summarized in Figure 13. The outcome suggests that there was little publication bias.

### 3.5. Dosage and Purity of Ligustrazine

Ligustrazine is one natural extract of ligustrazine. Ligustrazine hydrochloride was used in the intervention group of these 16 RCTs. The dosage of ligustrazine hydrochloride is 80 mg once daily. Calculated on the anhydrous basis, the purity of ligustrazine hydrochloride must not be any less than 99.0% [41]. Therefore, the strict pharmaceutical standardization makes the usage of ligustrazine evaluable.

## 4. Discussion

Ischaemic diseases can be improved by the so-called complementary medicine in some report [42]. Nevertheless, few relevant articles on ligustrazine for UA have been published in the English medical journals, and the situation reduces the evaluation of ligustrazine. Our study was designed to compare the efficacy and safety of ligustrazine preparations and conventional medicine by including 16 RCTs and 1356 participants. As shown above, there was a single study that mentioned the rate of cardiovascular events. Therefore, we were unable to summarize the effects of the routine use of antiangina treatment with ligustrazine on the reduction in incidence of acute myocardial infarction.

Nevertheless, the pooled analyses revealed that ligustrazine combined with conventional medicine appeared to have some benefits, such as increasing the rate of marked improvement of symptoms (RR = 1.24, 95% CI (1.18, 1.30)) and the rate of marked improvement of ECG (RR = 1.32, 95% CI (1.21, 1.45)) when compared with conventional Western medicine alone. Additionally, the use of ligustrazine was associated with significant trends in the reduction of the consumption of nitroglycerin (MD = −0.14, 95% CI (−0.20, −0.08)) and the level of fibrinogen (MD = −0.78, 95% CI (−0.91, −0.65)) when compared with conventional Western medicine alone. Furthermore, in the meta-analysis of these four outcomes, no statistical heterogeneity was noted among the comparisons (all* I* ^2^s = 0%). The outcomes of the time of the onset and the frequency of acute attack angina exhibited heterogeneity. Therefore, we should be careful in drawing conclusions about the efficiency of ligustrazine in the reduction of the time of onset or frequency of acute attack angina. There were no serious recorded adverse effects.

Although ligustrazine and conventional antiangina treatments that include ligustrazine exhibited some benefit, there are a number of limitations to this review. (1) The majority of the studies had small samples. (2) We only found and included Chinese studies. (3) The included studies were of low methodological quality and used neither blinding nor allocation concealment. (4) The duration of treatment was insufficient in the majority of the studies (14 days). Limitations still contribute enlightenment to future studies. Researchers can improve the methodology, such as allocation concealment, blinding method, treatment duration, and long-term follow-up. Well-designed trials of ligustrazine in UA management will promote its application correctly and our paper may stimulate appropriate evaluation on ligustrazine historically.

## 5. Conclusion

The addition of ligustrazine to conventional medicine possibly benefits unstable angina. However, quality evidence is needed to further assess its efficacy and safety.

## Figures and Tables

**Table 1:** Characteristics of included studies.

Studies	Sample (t/c)	Diagnosis standard	Age	Intervention group	Control group	Course (day)	Outcome measures
Guo 2007 [25]	20/16	ISFC/WHO	59 ± 6.7	Ligustrazine injection, nitrates, aspirin, ACE inhibitor	Nitrates, aspirin, ACE inhibitor	14	IAS, NIWAS, IECG, NIWECG

Liao and Luo 2006 [26]	34/34	ISFC/WHO	46–71	Ligustrazine injection, nitrates, aspirin	Nitrates and aspirin	15	IAS and NIWAS

Da 2008 [27]	38/36	CSC	41–83	Ligustrazine injection, nitrates, aspirin, Low Molecular Weight Heparin (LMWH), beta blockers	Nitroglycerin, aspirin, LMWH, beta blockers	14	IAS, NIWAS, IECG, NIWECG

Rong et al. 2001 [28]	32/32	Other	64.3 ± 7.2	Ligustrazine injection, nitrates, aspirin	Nitrates and aspirin	10	IAS and NIWAS

Peng 2014 [29]	43/42	CSC	61.2 ± 5.94	Ligustrazine injection, nitrates, antiplatelet drugs	Nitrates and antiplatelet drugs	14	IAS, NIWAS, IECG, NIWECG, AOT, SF, RNU, FIB

Wang and Hua 2004 [30]	30/28	ISFC/WHO	Unclear	Ligustrazine injection, beta blockers, aspirin, calcium channel blockers (CCB), LMWH, ACE inhibitor	Beta blockers, aspirin, CCB, LMWH, ACE inhibitor	28	IAS and NIWAS

Wei et al. 2012 [31]	60/60	CSC	48–79	Ligustrazine injection, antiplatelet drugs, nitrates, beta blockers	Antiplatelet drugs, nitrates, beta blockers	14	IAS, NIWAS, AOT, SF, FIB

Wang 2006 [32]	65/65	CSC	67.5 ± 9	Ligustrazine injection, beta blockers, nitrates, aspirin, CCB, ACE inhibitor	Beta blockers, nitrates, aspirin, CCB, ACE inhibitor	14	IAS and NIWAS

Zhou 2008 [33]	78/50	ISFC/WHO	39–89	Ligustrazine injection, nitroglycerin, aspirin, CCB, beta blockers	Nitroglycerin, aspirin, CCB, beta blockers	14	IAS and NIWAS

Li 2010 [34]	38/30	Other	42–76	Ligustrazine injection, nitroglycerin, aspirin, LMWH, beta blockers	Nitroglycerin, aspirin, LMWH, beta blockers	14	IAS and NIWAS

Sun 2007 [35]	48/48	Other	48–80	Ligustrazine injection, nitrates, aspirin, beta blockers, statins, ACE inhibitor	Nitrates, aspirin, beta blockers, statins, ACE inhibitor	21	IAS, NIWAS, IECG, NIWECG, FIB

Wang 2008 [36]	42/42	ISFC/WHO	48–78	Ligustrazine injection, nitrates, aspirin, beta blockers, CCB, statins, ACE inhibitor	Nitrates, aspirin, beta blockers, CCB, statins, ACE inhibitor	14	IAS, NIWAS, IECG, NIWECG

Fan 2014 [37]	63/63	Other	73.5 ± 12.6	Ligustrazine injection, nitrates, aspirin, statins	Nitrates, aspirin, statins, beta blockers	14	IAS, NIWAS, IECG, NIWECG, AOT, SF, RNU

Fu and Lin 2009 [38]	40/41	Other	Unclear	Ligustrazine injection, nitrates, aspirin, statins, LMWH, beta blockers	Nitrates, aspirin, statins, LMWH, beta blockers	14	IAS and NIWAS

Meng et al. 2005 [39]	38/38	ISFC/WHO	68.8 ± 7.8	Ligustrazine injection, nitrates, beta blockers, ACE inhibitor, CCB, LMWH	Nitrates, beta blockers, ACE inhibitor, CCB, LMWH	10	IAS, NIWAS, IECG, NIWECG, FIB, CEs

Wang et al. 2005 [40]	32/30	ISFC/WHO	50–66	Ligustrazine injection, nitrates, aspirin, LMWH, beta blockers	Nitrates, aspirin, LMWH, beta blockers	14	IAS, NIWAS, IECG, NIWECG

**Table 2:** Methodological quality of the included studies.

Studies	Random sequence generation	Allocation concealment	Blinding of participants and personnel	Blinding of outcome assessment	Incomplete outcome data	Selective reporting	Other bias
Guo 2007 [25]	Low risk	Unclear	Low risk	Low risk	Low risk	Low risk	Low risk
Liao and Luo 2006 [26]	Low risk	Unclear	Unclear	Unclear	Low risk	Low risk	Low risk
Da 2008 [27]	Low risk	Unclear	Unclear	Unclear	Low risk	Low risk	Low risk
Rong et al. 2001 [28]	Low risk	Unclear	Unclear	Unclear	Low risk	Low risk	Low risk
Peng 2014 [29]	Low risk	Unclear	Unclear	Unclear	Low risk	Low risk	Low risk
Wang and Hua 2004 [30]	Low risk	Unclear	Unclear	Unclear	Low risk	Low risk	Low risk
Wei et al. 2012 [31]	Low risk	Unclear	Unclear	Unclear	Low risk	Low risk	Low risk
Wang 2006 [32]	Low risk	Unclear	Unclear	Unclear	Low risk	Low risk	Low risk
Zhou 2008 [33]	Low risk	Unclear	Unclear	Unclear	Low risk	Low risk	Low risk
Li 2010 [34]	Low risk	Unclear	Unclear	Unclear	Low risk	Low risk	Low risk
Sun 2007 [35]	Low risk	Unclear	Unclear	Unclear	Low risk	Low risk	Low risk
Wang 2008 [36]	Low risk	Unclear	Unclear	Unclear	Low risk	Low risk	Low risk
Fan 2014 [37]	Low risk	Unclear	Unclear	Unclear	Low risk	Low risk	Low risk
Fu and Lin 2009 [38]	Low risk	Unclear	Unclear	Unclear	Low risk	Low risk	Low risk
Meng et al. 2005 [39]	Low risk	Unclear	Unclear	Unclear	Low risk	Low risk	Low risk
Wang et al. 2005 [40]	Low risk	Unclear	Unclear	Unclear	Low risk	Low risk	Low risk
